# An Impressive Finding of Sarcoid-Like Reaction to Tattooing

**DOI:** 10.7759/cureus.35401

**Published:** 2023-02-24

**Authors:** Alexandra K Mathis, Beverly A Johnson

**Affiliations:** 1 Medicine, Edward Via College of Osteopathic Medicine, Auburn, USA; 2 Dermatology, University of Florida College of Medicine, Gainesville, USA

**Keywords:** sarcoid-like reaction, sarcoidosis-like, cosmetic tattoo, tattoo pigment, cutaneous sarcoidosis

## Abstract

This case represents an impressive finding of a young male who developed cutaneous sarcoid-like reactions that arose secondarily to tattoo ink. Cutaneous manifestations of sarcoidosis can present themselves in various ways, be caused by different inducing factors, and may present themselves not only cutaneously but as one of the many findings of systemic sarcoidosis. A 24-year-old black gentleman presented to the dermatology clinic with a manifestation of papules in his tattoos that covered a majority of his body. The patient tried hypoallergenic tattoo ink to see if this would prevent the formation of these bumps; however, this only further provoked the production of these papules confined to tattooed areas. Skin findings revealed linear raised papules in a continuous fashion in areas where the patient had tattoo ink on his torso, bilateral arms and legs, and face. At presentation, he denied any constitutional, pulmonary, or ophthalmologic symptoms. Pathological findings revealed sarcoidal granulomatous dermatitis showing nodular non-necrotizing granulomatous inflammatory infiltrate involving the superficial and deep dermis. The patient was then evaluated by pulmonology to rule out any systemic findings of sarcoidosis, which showed a negative chest x-ray.

The patient was started on oral prednisone and topical pimecrolimus cream, and when he returned for his one-month follow-up, there were minimal to no visible cutaneous lesions. Tattoo ink has been shown to cause a variety of cutaneous reactions such as infections, neoplasms, and inflammatory dermatoses such as eczema, lichen planus, psoriasis, vitiligo, and sarcoidosis. Cutaneous sarcoid-like reactions secondary to tattoo ink are rare findings; however, they must be taken seriously due to the risk of systemic sarcoidosis involvement both at initial presentation and in the future.

## Introduction

Sarcoidosis is classically hallmarked by noncaseating granulomas of unknown etiology that traditionally occur systemically, with lung involvement being the most common. However, sarcoidosis can involve almost any organ system including the skin. Cutaneous manifestations of sarcoidosis include erythema nodosum, lupus pernio, and papular or plaque lesions [1]. Cutaneous sarcoidosis may occur as one of the many findings of systemic sarcoidosis or occur secondarily to a precipitating factor such as scars, infections, or even tattoo ink [1]. Tattoo pigment can precipitate inflammatory states, causing a wide array of cutaneous reactions; however, granulomatous reactions, as seen in cutaneous sarcoidosis, are rare [2,3].

## Case presentation

A 24-year-old black gentleman presented to the dermatology clinic with concerns of bumps in his tattoos that covered a majority of his body. He started developing papules, all confined to tattooed areas, about five years after getting his tattoos. As a professional boxer, he believed that the dimensionality of his tattoos set him apart from his boxing peers. When questioning the tattoo artist as to why he developed these bumps, he was assured by the tattoo artist that the problem was “just the ink” and suggested the use of “hypoallergenic ink” instead. The patient agreed to have more tattoos done in new areas; however, a few months after getting the tattoos, he began to see more papules develop in spite of the “hypoallergenic ink.” The patient then decided to visit a dermatologist for a second opinion.

The patient is a healthy and physically fit young male with no significant past medical history. His family’s past medical history was also noncontributory. He denied having any skin conditions or problems prior to the tattoo ink. The patient is a professional boxer who is married and has two children.

**Figure 1:**
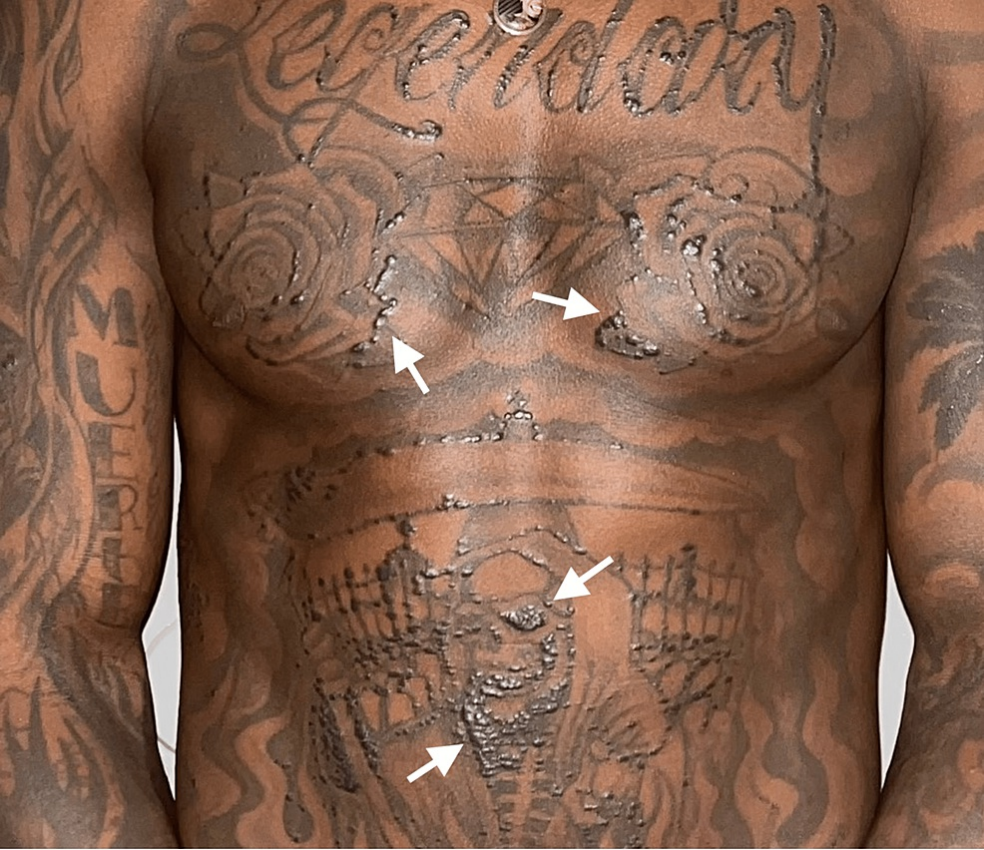
Dermatologic examination at initial presentation showing linear raised papules confined to the patient’s tattoo on the torso

On presentation, skin findings revealed linear raised papules in a continuous fashion in areas where the patient had tattoo ink on his torso, bilateral arms and legs, and face (Figure 1). At his initial visit, he denied any constitutional, pulmonary, or ophthalmologic symptoms.

A punch biopsy was performed on his right forearm, which revealed sarcoidal granulomatous dermatitis showing nodular non-necrotizing granulomatous inflammatory infiltrate involving the superficial and deep dermis. There was granular black pigment consistent with tattoo ink within the granulomas. The patient’s diagnosis of sarcoidal reaction secondary to tattooing was confirmed. A chest x-ray was ordered to rule out any systemic findings of sarcoidosis, which did not show any hilar adenopathy.

**Figure 2:**
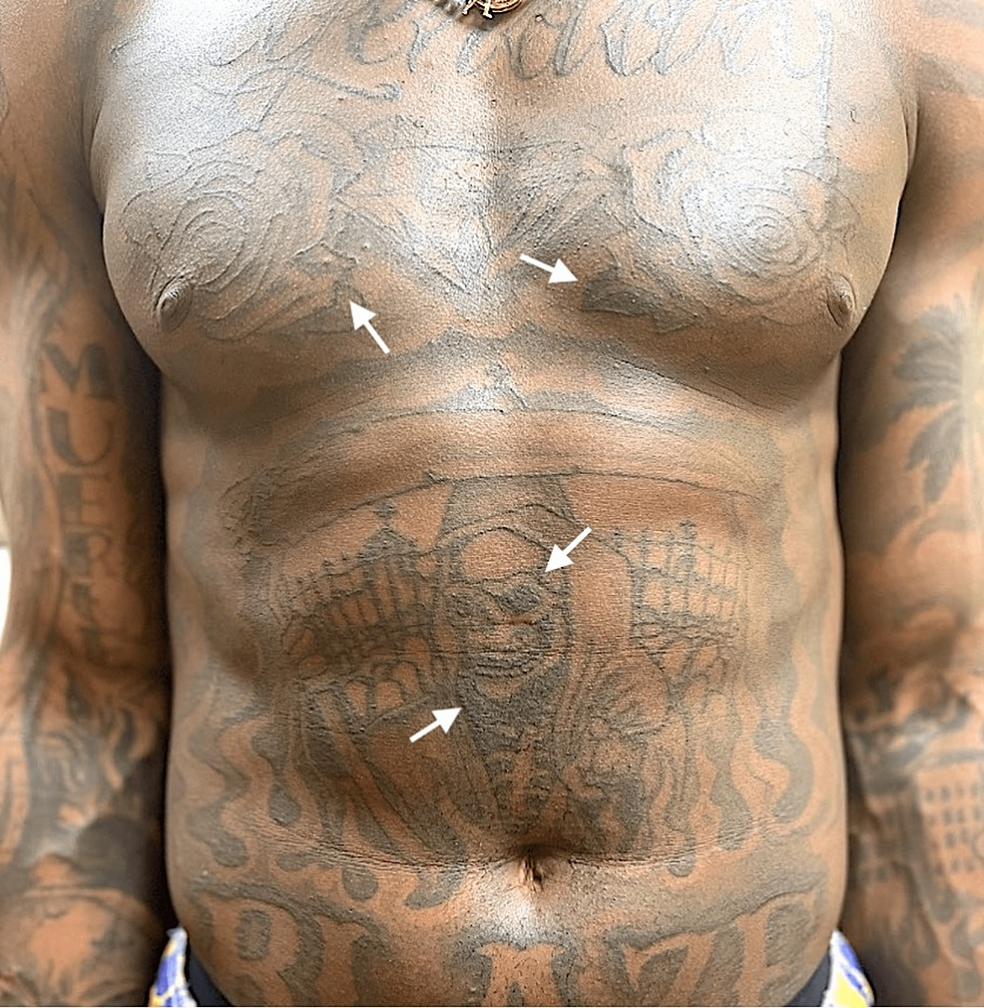
Dermatologic examination at one-month follow-up showing resolution of sarcoid-like lesions on the torso

The patient was started on oral prednisone 40 mg daily for two weeks and given topical pimecrolimus 1% cream. When the patient returned for his monthly follow-up, there were minimal to no visible cutaneous lesions on his trunk, arms, legs, or face (Figure 2). The treatment regimen we chose targeted these lesions without affecting any of his tattoos, leaving the patient very pleased with the results.

## Discussion

This case represents an impressive finding of cutaneous sarcoid-like reactions that arose secondarily to tattoo ink that covered most of his body. Cutaneous sarcoidosis can present itself in various ways, is caused by different initiating factors, and may present itself not only cutaneously but as one of the many findings of systemic sarcoidosis [2,4]. When identifying and treating patients with initial cutaneous manifestations of sarcoidosis, it is important to investigate the potential for systemic findings as well [5]. In our patient, no hilar adenopathy was seen on the chest x-ray, a finding typically seen in systemic sarcoidosis.

Currently, there is no clear-cut delineation histopathologically between cutaneous sarcoidosis and sarcoid-like reactions secondary to a foreign body, in this case, the tattoo ink. As our patient did not have any systemic findings of sarcoidosis and insignificant past medical history, we believe that this case presentation represents a sarcoid-like reaction due to tattoo ink instead of a cutaneous presentation of sarcoidosis [5].

These sarcoid-like lesions were not immediately present. Instead, some developed over months, while others took years to develop. This was also seen in a case report published in 2005 in the *Journal of the American Academy of Dermatology* (*JAAD*) where the patient developed noticeable changes in her tattoo almost a year later [6]. However, in that case study, the patient also developed an allergy to metal in the pigment of the tattoo, specifically nickel, whereas our patient did not have any symptoms of an allergy to the metal in his chain necklace [6].

Our patient was managed on oral steroids and topical pimecrolimus cream, which resulted in the resolution of his skin finding without damage to his tattoos, which was a major concern for the patient. According to UpToDate, oral steroids are currently the standard of care for rapidly progressive sarcoidosis with marked improvement occurring within three to four weeks, which is consistent with the timeline of treatment for our patient [7]. Other treatment options include topical and intralesional corticosteroids, cryotherapy, and antimalarial and immunosuppressive drugs. A study published in 2002 in the *British Journal of Dermatology* reported a patient who had unsuccessful treatment of cutaneous sarcoid with topical and oral steroids but was responsive to topical tacrolimus, an immunosuppressive drug similar in safety profile to pimecrolimus [8]. Another study reported a patient with cutaneous sarcoidosis who tried several treatments including repeated cryotherapy, topical vitamin E, and intralesional and topical corticosteroids with a lack of clinical improvement but did have a resolution of dermatologic findings with topical photodynamic therapy [9]. In our patient, he had resolution of the cutaneous lesions with treatment using only oral prednisone 40 mg daily for two weeks and topical pimecrolimus 1% cream.

Tattoo ink has been shown to cause a variety of reactions such as infections, neoplasms, and inflammatory dermatoses. Inflammatory dermatoses include eczema, lichen planus, psoriasis, vitiligo, and even cutaneous sarcoidosis [2,10]. Although cutaneous sarcoid-like reactions secondary to tattoo ink are rare findings, they are important to be attentive to due to the risk of systemic sarcoidosis involvement both at initial presentation and in the future [5,11,12].

## Conclusions

We, the authors, believe that the incidence of sarcoid-like reactions and other cutaneous findings secondary to tattoo ink may increase due to the increasing popularity of tattoos and diminished negative connotations associated with tattooing. So, it may be of benefit to educate tattoo artists on their clients developing these sarcoid-like reactions and other skin reactions secondary to tattoo ink in order to better serve their current and future clientele.
